# Antimicrobial resistance and molecular epidemiological typing of *Neisseria gonorrhoeae* isolates from Kyrgyzstan in Central Asia, 2012 and 2017

**DOI:** 10.1186/s12879-021-06262-w

**Published:** 2021-06-12

**Authors:** Saliya Karymbaeva, Iryna Boiko, Susanne Jacobsson, Galina Mamaeva, Ainagul Ibraeva, Dilara Usupova, Daniel Golparian, Magnus Unemo

**Affiliations:** 1 WHO Collaborating Centre for Gonorrhoea and Other STIs, National Reference Laboratory for STIs, Department of Laboratory Medicine, Clinical Microbiology, Faculty of Medicine and Health, Örebro University Hospital, SE-701 85 Örebro, Sweden; 2 Department of Functional and Laboratory Diagnostics, I. Horbachevsky Ternopil National Medical University, Ternopil, Ukraine; 3 Republican Dermatovenerological Centre, Bishkek, Kyrgyzstan

**Keywords:** *Neisseria gonorrhoeae*, Gonorrhoea, Antimicrobial resistance (AMR), Surveillance, NG-MAST, Ceftriaxone, Cefixime, Azithromycin, Kyrgyzstan

## Abstract

**Background:**

Gonorrhoea and antimicrobial resistance (AMR) in *Neisseria gonorrhoeae* are significant public health concerns globally. Nearly no gonococcal AMR data are available from Central Asia, and no data from Kyrgyzstan has been published. We examined, for the first time, AMR and molecular epidemiology of *N. gonorrhoeae* isolates cultured in Kyrgyzstan in 2012 and 2017, in order to inform refinements of the Kyrgyz national gonorrhoea management guidelines.

**Methods:**

*N. gonorrhoeae* isolates cultured in 2012 (*n* = 84) and 2017 (*n* = 72) in Kyrgyzstan were examined. MICs of nine antimicrobials were determined using Etest and, where available, clinical breakpoints from the EUCAST were applied. *N. gonorrhoeae* multiantigen sequence typing (NG-MAST) was also performed.

**Results:**

The overall resistance levels were high to ciprofloxacin (88.5%), tetracycline (56.9%), benzylpenicillin (39.1%), and kanamycin (4.7%). Resistance to cefixime (0.6%, *n* = 1 isolate), azithromycin (0.6%, n = 1), and gentamicin (0.6%, n = 1) was rare. No resistance to ceftriaxone or spectinomycin was found. However, the proportion of isolates with decreased susceptibility (MIC = 0.125 mg/L) to ceftriaxone and cefixime was 12.8 and 11.5%, respectively. Gonococcal isolates were assigned 69 sequence types, of which 52 (75.4%) were new.

**Conclusions:**

The gonococcal population in Kyrgyzstan in 2012 and 2017 showed a high genetic diversity. Ceftriaxone, 500–1000 mg, in combination with azithromycin 2 g or doxycycline, particularly when chlamydial infection has not been excluded, should be recommended as empiric first-line treatment. Spectinomycin 2 g could be an alternative treatment, and given with azithromycin 2 g if pharyngeal gonorrhoea has not been excluded. Fluoroquinolones, aminoglycosides, benzylpenicillin, or tetracyclines should not be used for empiric treatment of gonorrhoea in Kyrgyzstan. Timely updating and high compliance to national gonorrhoea treatment guidelines based on quality-assured AMR data is imperative. Expanded and improved gonococcal AMR surveillance in Kyrgyzstan is crucial.

## Background

Gonorrhoea, caused by *Neisseria gonorrhoeae*, is a significant public health concern globally. In 2016, the World Health Organization (WHO) estimated 87 million new cases of gonorrhoea among adults worldwide, which places gonorrhoea as the second most common bacterial sexually transmitted infection (STI), i.e., after *Chlamydia trachomatis* infections [1]. *N. gonorrhoeae* has developed antimicrobial resistance (AMR) to all drugs previously used for the treatment of gonorrhoea. Currently, ceftriaxone is the only remaining effective option for empiric first-line therapy, which is frequently given together with azithromycin in dual therapies [2–16]. However, treatment failures with ceftriaxone, including one with ceftriaxone plus azithromycin dual therapy [13] have been confirmed in many countries [4]. Furthermore, since 2015 an internationally spreading ceftriaxone-resistant strain has been detected in many countries worldwide [16–23] and the first strain with ceftriaxone resistance combined with high-level resistance to azithromycin was detected in both the United Kingdom and Australia in 2018 [14, 15, 24]. As stressed in the WHO global action plan [25] and the European response plan [26], it is essential to substantially enhance the quality assured surveillance of gonococcal AMR worldwide. Molecular epidemiological typing of gonococci can effectively support the gonococcal AMR surveillance and *N. gonorrhoeae* multiantigen sequence typing (NG-MAST) has been used in many countries globally [27–33].

Gonococcal infections acquired in, or from, Asia represent most of the confirmed treatment failures with ceftriaxone, and several ceftriaxone-resistant strains appear to have emerged in Asia and subsequently spread globally [4, 34, 35]. In the Kyrgyz Republic (Kyrgyzstan), Central Asia, gonorrhoea is a mandatorily reported infection, which incidence has decreased during the recent decades. The outdated 2005 Kyrgyz national gonorrhoea management guideline recommended treatment with ciprofloxacin 500 mg single oral dose, ceftriaxone 250 mg intramuscular (IM) dose, cefixime 400 mg single oral dose, or spectinomycin 2 g IM; and kanamycin 2 g IM was recommended as an alternative treatment. However, the 2012 Kyrgyz national gonorrhoea management guideline, in which the initial AMR results of the present study were taken into account, recommended dual therapy for gonococcal urethritis: ceftriaxone 250 mg IM or spectinomycin 2 g IM together with doxycycline 100 mg orally 2 times per day for 7 days or azithromycin 1 g single oral dose; and cefotaxime 500 mg IM or cefixime 400 mg single oral dose was recommended as alternative treatment [36]. Since 2014, the Ministry of Health of the Kyrgyz Republic is additionally suggesting monotherapy with ceftriaxone 250 mg IM or spectinomycin 2 g IM for syndromic management; alternative treatment with cefixime 400 mg single oral dose [37]. In Kyrgyzstan, the majority of STI cases are not treated by dermatovenereologists due to reorganisation of the dermatovenerogical services and reforming of the health system, but by primary care physicians, urologists and gynaecologists, mainly private health care providers. In general, very limited gonococcal AMR data is available in the Eastern part of the WHO European Region (i.e. mostly countries earlier belonging to the Soviet Union). Accordingly, limited gonococcal AMR data have only been reported from Russia [33, 38–41], Belarus [42–44], and Ukraine [45, 46]. No AMR or phenotypic/genetic characteristics of *N. gonorrhoeae* strains spreading in Kyrgyzstan have been internationally published.

The aims of the present study were to, for the first time, describe AMR to previous and current treatment options and molecular epidemiology, by means of NG-MAST, of *N. gonorrhoeae* isolates cultured in Kyrgyzstan (2012 and 2017). Our quality-assured gonococcal AMR data were used to inform refinements of the 2012 Kyrgyz national gonorrhoea management guideline [36].

## Methods

### Gonorrhoea patients and *N. gonorrhoeae* culture

*N. gonorrhoeae* culture-positive patients with urogenital symptoms (mainly urethral or vaginal discharge) attending the out-patient clinic of the Republican Dermatovenereological Centre, Bishkek, Kyrgyzstan in 2012 and in 2017 were enrolled in the study. Informed consent was obtained from all included patients and demographic data (sex and age) collected. Exclusion criteria were: i) no verified gonococcal culture and ii) complicated STI. No patient identification information was available in the study. All patients were to be managed in accordance with the Kyrgyz national gonorrhoea management guideline [36], which was updated based on the AMR results of the present study.

*N. gonorrhoeae* isolates were obtained from urethral samples from males and cervical samples from females cultured on selective GCVIT agar plates (3.6% Difco GC Medium Base agar (BD, Diagnostics, Sparks, MD, USA), supplemented with 1% IsoVitalex (BD, Diagnostics, Sparks, MD, USA) and 1% VCNT inhibitor (BD, Diagnostics, Sparks, MD, USA)) incubated in candle jars with extra humidity at 36 ± 1 °C. All isolates were confirmed as *N. gonorrhoeae* by identification of typical colonies on the selective GCVIT agar plates, Gram-negative diplococci in microscopy, rapid oxidase reaction, and the PhadeBact GC Monoclonal test (Bactus AB, Solna, Sweden), and subsequently preserved as previously described [47]. All examined gonococcal isolates were cultured as part of the routine diagnostics (standard care) [36].

### Antimicrobial susceptibility testing

The minimum inhibitory concentration (MIC; mg/L) of ceftriaxone, cefixime, spectinomycin, azithromycin, ciprofloxacin, benzylpenicillin, tetracycline, gentamicin, and kanamycin were determined using the Etest method (bioMérieux, Marcy-l’Etoile, France), according to the instructions from the manufacturer. All results were interpreted using whole MIC doubling dilutions and, where available, current clinical breakpoints for susceptibility (S) and resistance (R) according to The European Committee on Antimicrobial Susceptibility Testing (EUCAST [48]]. For azithromycin, EUCAST does not recommend any clinical breakpoints, and the EUCAST azithromycin epidemiological cut-off value (ECOFF) of MIC> 1 mg/L [48] was used to indicate isolates with azithromycin resistance determinants (referred to as resistant hereafter). Previously published interpretative criteria were used for gentamicin [49] and kanamycin [50, 51]. MIC_90_ and MIC_50_ values were defined as the lowest concentration of the antimicrobial at which 90% and 50% of the isolates were inhibited, respectively. β-lactamase production was identified using a Nitrocefin test (Oxoid, Basingstoke, England). The 2008 WHO *N. gonorrhoeae* reference strains [52] were used for quality controls of all phenotypic and molecular characterisation.

### Isolation of genomic DNA

Bacterial DNA was isolated in the robotised NorDiag Bullet instrument (NorDiag ASA Company, Oslo, Norway) using the BUGS’n BEADS™ STI-*fast* kit (NorDiag ASA Company, Oslo, Norway), according to the instructions from the manufacturer.

### Molecular epidemiological typing

Molecular epidemiological typing by means of NG-MAST [27] was performed for all viable *N. gonorrhoeae* isolates (*n* = 143), as previously described [52]. NG-MAST allele numbers of the more variable segments of *porB* and *tbpB*, and sequence types (STs) were assigned using the NG-MAST website (www.ng-mast.net).

### Statistical analysis

Statistical analysis was performed using the MedCalc Statistical Software v 19.3.1 (MedCalc Software bvba, Ostend, Belgium). The 95% confidence interval (95% CI) was calculated using the exact binominal distribution method. Z-test, Fisher exact and Mann-Whithey U tests, odds ratio (OR) were used for comparison between groups, as appropriate. The level of significance was set at P<0.05.

## Results

### Gonorrhoea patients and *Neisseria gonorrhoeae* isolates

In total, 156 *N. gonorrhoeae* isolates were cultured from 156 patients (146 males (93.6%) and 10 females (6.4%)) attending the out-patient clinic of the Republican Dermatovenereological Centre, Bishkek, Kyrgyzstan, i.e., 84 isolates in 2012 and 72 isolates in 2017. The mean age for the males was 25.7 years (median age: 24 years; range: 18–59 years; IQR = 21–27) and for the females 26 years (median age: 26 years; range: 23–32 years; IQR = 25–27).

### Antimicrobial susceptibility of *N. gonorrhoeae* isolates (*n* = 156) in Kyrgyzstan

The results of the antimicrobial susceptibility testing of all isolates are summarised in Table 1.

**Table 1:** Antimicrobial susceptibility of *Neisseria gonorrhoeae* isolates (*n* = 156) from Bishkek, Kyrgyzstan, 2012 (*n* = 84) and 2017 (*n* = 72)

		2012 (*n* = 84)			2017 (*n* = 72)			Total (*n* = 156)		
		S	I^a^	R^b^	S	I^a^	R^b^	S	I^a^	R^b^
CRO	N	84	NA	0	72	NA	0	156	NA	0
	% (95% CI)	100 (95.7–100)	NA	0 (0–4.3)	100 (95.0–100)	NA	0 (0–5.0)	100 (97.7–100)	NA	0 (0–2.3)
CFM	N	84	NA	0	71	NA	1	155	NA	1
	% (95% CI)	100 (95.7–100)	NA	0 (0–4.3)	98.6 (92.5–100)	NA	1.4 (0–7.5)	99.4 (96.6–100)	NA	0.6 (0.01–3.5)
SPC	N	84	NA	0	72	NA	0	156	NA	0
	% (95% CI)	100 (95.7–100)	NA	0 (0–4.3)	100 (95.0–100)	NA	0 (0–5.0)	100 (97.7–100)	NA	0 (0–2.3)
AZM	N	83	NA	1	72	NA	0	155	NA	1
	% (95% CI)	98.8 (93.5–100)	NA	1.2 (0.03–6.5)	100 (95–100)	NA	0 (0–5.0)	99.4 (96.6–100)	NA	0.6 (0.01–3.5)
CIP	N	12	0	72	5	1	66	17	1	138
	% (95% CI)	14.3 (7.6–23.6)	0 (0–4.3)	85.7 (76.4–92.4)	6.9 (2.3–15.4)	1.4 (0.04–7.5)	91.7 (82.8–96.9)	10.9 (6.5–16.9)	0.6 (0.01–3.5)	88.5 (82.4–93.1)
PEN	N	6	52	26	3	34	35	9	86	61
	% (95% CI)	7.1 (2.6–14.9)	61.9 (50.7–72.3)	31.0 (21.4–42.0)	4.2 (0.9–11.7)	47.2 (35.3–59.3)	48.6 (36.6–60.7)	5.8 (2.7–10.7)	55.1 (46.9–63.1)	39.1 (31.4–47.2)
TET	N	ND	ND	ND	18	13	41	Data available only in 2017		
	% (95% CI)	ND	ND	ND	25 (15.5–36.6)	18.1 (10.0–29.0)	56.9 (44.7–68.5)			
KAN	N	47	33	4	ND	ND	ND	Data available only in 2012		
	% (95% CI)	56 (44.7–66.8)	39.3 (28.8–50.6)	4.7 (1.3–11.7)	ND	ND	ND			
GEN	N	83	1	0	69	2	1	152	3	1
	% (95% CI)	98.8 (93.5–199)	1.2 (0.03–6.5)	0 (0–4.3)	95.8 (88.3–99.1)	2.8 (0.4–9.7)	1.4 (0.04–7.5)	97.4 (93.5–99.3)	2 (0.4–5.6)	0.6 (0.01–3.5)
		*n* = 75			*n* = 72			*n* = 147		
		Negative/Positive								
PPNG	N	60	15		55	17		115	32	
	% (95% CI)	80 (69.2–88.4)	20 (11.7–30.8)		76.4 (64.9–85.6)	23.6 (14.4–35.1)		78.2 (70.7–84.6)	21.8 (15.4–29.4)	

S, susceptible; I, susceptible, increased exposure; R, resistant; CRO, ceftriaxone; CFM, cefixime; AZM, azithromycin; SPC, spectinomycin; CIP, ciprofloxacin; PEN, benzylpenicillin; TET, tetracycline; KAN, kanamycin; PPNG, penicillinase-producing *Neisseria gonorrhoeae*; n, number of isolates; CI, confidence interval; NA, not applicable; ND, Not determined

^a^A microorganism is categorised as “Susceptible, Increased exposure” when there is a high likelihood of therapeutic success because exposure to the agent is increased by adjusting the dosing regimen or by its concentration at the site of infection (www.eucast.org/newsiandr/)

^b^The breakpoints (susceptible/resistant) from the EUCAST [48] were as follows: ceftriaxone and cefixime (MIC≤0.125/> 0.125 mg/L), azithromycin (MIC≤1/> 1 mg/L), spectinomycin (MIC≤64/> 64 mg/L), ciprofloxacin (MIC≤0.032/> 0.064 mg/L), benzylpenicillin (MIC≤0.064/> 1.0 mg/L), and tetracycline (MIC≤0.5/> 1.0 mg/L). Previously published breakpoints were used for gentamicin (MIC≤4/> 16 mg/L [49]) and kanamycin (MIC≤4/≥16 mg/L) [50, 51]

Briefly, the overall proportions of isolates with higher levels of resistance were as follows: ciprofloxacin – 88.5%, tetracycline - 56.9%, benzylpenicillin – 39.1%, and kanamycin - 4.7%. Resistance to cefixime, azithromycin, and gentamicin was detected in one isolate each (0.6%). The susceptibility to all these antimicrobials, except azithromycin, declined from 2012 to 2017. No isolates resistant to ceftriaxone or spectinomycin were identified. The overall proportion of penicillinase-producing *N. gonorrhoeae* (PPNG) isolates was 21.8%, and the proportion of PPNG was stable in 2012 and 2017 (*P* = 0.6) (Table 1).

Despite that no isolates resistant to ceftriaxone (MIC> 0.125 mg/L) were identified, the MIC_90_ to both ceftriaxone and cefixime was 0.125 mg/L, which is exactly at the clinical breakpoint for resistance. The overall numbers of isolates with MIC = 0.125 mg/L were 12.8% (20/156) and 11.5% (18/156) for ceftriaxone and cefixime, respectively. Nevertheless, the proportion of isolates with extended-spectrum cephalosporin (ESC) MIC = 0.125 mg/L was not significantly different in 2012 and 2017 (*P* = 0.07).

### Molecular epidemiological characterisation (NG-MAST)

In total, 69 different NG-MAST STs (14 NG-MAST genogroups) were found among the 143 sequenced isolates. Majority of STs (75.4%, 52/69) had not been previously described. The most common STs were ST1751 (14.7%, *n* = 21), ST15952 (4.9%, *n* = 7), ST368 (4.2%, *n* = 6), ST1691 (4.2%, n = 6), ST569 (3.5%, *n* = 5), ST807 (3.5%, n = 5), and ST15951 (2.8%, *n* = 4). Eight different STs (11.6%, 8/69) represented by three isolates each were found: ST387, ST972, ST10687, ST14617, ST15955, ST15957, ST15961, and ST15981. Ten STs (14.5%, 10/69) included two isolates each: ST157, ST5941, ST15953, ST15954, ST15958, ST15959, ST15960, ST15968, ST15986, and ST15989. Forty-four STs (63.8%, 44/69) were represented by a single isolate.

The most common NG-MAST genogroups were G1751 (14.7%, *n* = 21), G5488 (8.4%, *n* = 12), G436 (5.6%, *n* = 8), G386 (4.2%, *n* = 6), G569 (3.5%, *n* = 5), G972 (2.8%, *n* = 4), and G387 (2.1%, *n* = 3). Two genogroups included two isolates each. Notably, seven isolates (4.9%, 7/143) belonged to several previously described genogroups: G807/G228/G51/G25 (3.5%, n = 5) and G2862/G1105 (1.4%, *n* = 2). Seventy-two isolates (50.3%, 72/143) were non-groupable. The most common NG-MAST genogroups and their MICs of ESCs are summarised in Table 2.

**Table 2:** *Neisseria gonorrhoeae* multiantigen sequence typing (NG-MAST) genogroups, sequence types, and minimum inhibitory concentrations (MICs, mg/L) of ceftriaxone and cefixime for *Neisseria gonorrhoeae* (*n* = 143) isolated in Bishkek, Kyrgyzstan, 2012 and 2017

NG-MAST (number of isolates)			Number of isolates with MIC (mg/L):								
Genogroups	Sequence types	ESC	≤0.016	0.023	0.032	0.047	0.064	0.094	0.125	0.25	0.5
1751 (21)	1751 (21)	CFM	20		1						
		CRO	18	1	1		1				
5488 (12)	15,952 (7), 10,687 (3), 1597 (1), 15,990 (1)	CFM	10	1				1			
		CRO	11					1			
436 (8)	1691 (6), 15,964 (1), 15,997 (1)	CFM	8								
		CRO	6			2					
368 (6)	368 (6)	CFM	4			1			1		
		CRO	5						1		
569 (5)	569 (5)	CFM	2	1	1				1		
		CRO	3						2		
807/228/51/25 (5)	807 (5)	CFM	5								
		CRO	3						2		
972 (4)	972 (3), 2870 (1)	CFM	3						1		
		CRO	3						1		
387 (3)	387 (3)	CFM	2						1		
		CRO	2						1		
340 (2)	340 (1), 15,992 (1)	CFM	1	1							
		CRO	1	1							
807 (2)	5941 (2)	CFM	1						1		
		CRO	1						1		
2862/1105 (2)	2862 (1), 15,979 (1)	CFM	2								
		CRO	2								
Non-grouped (72): 15951 (4), 14,617 (3), 15,955 (3), 15,957 (3), 15,961 (3), 15,981 (3), 157 (2), 15,953 (2), 15,954 (2), 15,958 (2), 15,959 (2), 15,960 (2), 15,968 (2), 15,986 (2), 15,989 (2); STs presented by single isolate (35)		CFM	58				1	3	9		1
		CRO	58	1	1	3			9		

NG-MAST, *Neisseria gonorrhoeae* multiantigen sequence typing; MIC, minimum inhibitory concentration; ESC, extended-spectrum cephalosporins; CFM, cefixime; CRO, ceftriaxone

Of all isolates with in vitro decreased susceptibility (MIC = 0.125 mg/L) to ceftriaxone (*n* = 17) and cefixime (*n* = 16), fourteen (82.3 and 87.5%, respectively) were viable at reculture prior to NG-MAST. These isolates belonged to 13 different NG-MAST STs: 15986 (*n* = 2) and 12 STs comprising single isolate (ST368, ST387, ST569, ST5941, ST2870, ST1318, ST14617, ST15949, ST15960, ST15974, ST15988, and ST15999). Notably, the single isolate resistant to azithromycin (MIC = 6 mg/L) belonged to ST15952 (G5488), while the single isolate resistant to cefixime (MIC = 0.5 mg/L) was assigned as ST15999 (non-groupable). No isolate belonged to the internationally spreading ST1407 [28, 30, 32].

There was a significant association between ST15952 and resistance to benzylpenicillin (OR = 0.11; 95% CI 0.013–0.92; *P* = 0.04); ST14617 and PPNG (OR = 0.04; 95% CI 0.002–0.8; *P* = 0.03); and ST15986 and decreased susceptibility to ESC, i.e. MIC = 0.125 mg/L (OR = 0.03; 95% CI 0.001–0.6; P = 0.03). No other association between ST and antimicrobial susceptibility was found, and no STs were specific for sex or age group.

## Discussion

The present study describes the first gonococcal AMR data and molecular characteristics of *N. gonorrhoeae* isolates (obtained in 2012 and 2017) in Kyrgyzstan.

High prevalence of resistance was observed for previous international first-line antimicrobials such as ciprofloxacin (88.5%), tetracycline (56.9%), and benzylpenicillin (39.1%). Overall, 21.8% of the gonococcal isolates in Kyrgyzstan were PPNG strains, which cause high-level resistance to benzylpenicillin. Similar high levels of resistance to ciprofloxacin, tetracycline and benzylpenicillin have been described in many other countries in Europe and worldwide [2–7, 25], and none of those antimicrobials can be recommended for empiric first-line therapy of gonorrhoea in Kyrgyzstan as well as in most other countries globally. Our initial AMR results from 2012 formed the basis for refining the national gonorrhoea management guideline in Kyrgyzstan, i.e. to exclude ciprofloxacin and cefixime as recommended empiric first-line treatments and kanamycin as an alternative treatment [36]. Resistance to kanamycin was detected in 4.7% of isolates, but resistance to cefixime, azithromycin and gentamicin only in one isolate each (0.6%). Fortunately, no isolates were resistant to ceftriaxone or spectinomycin. However, 12.8 and 11.5% of the gonococcal isolates in Kyrgyzstan showed an MIC of 0.125 mg/L for ceftriaxone and cefixime, respectively. Gonococcal isolates with ESC MICs of 0.125 mg/L have previously resulted in gonorrhoea treatment failure, particularly in pharyngeal infection, with both cefixime and ceftriaxone [4, 35, 53]. Accordingly, these isolates can be considered to have a decreased in vitro susceptibility to ESCs. Notably, only one Kyrgyz isolate (0.6%) was resistant to azithromycin, which is frequently given together with ceftriaxone in the internationally recommended dual treatments of gonorrhoea [7, 9–11]. The increasing resistance to azithromycin internationally is challenging the future inclusion of azithromycin in these dual therapies [3, 4]. Currently, it is also a concern that the resistance to several antimicrobials, including azithromycin, may increase due to the widespread use during the COVID-19 pandemic [54]. Consequently, it is crucial to survey the spread of AMR gonococcal strains and MIC increases, continuously or at a minimum in regular periodic surveys, with special emphasis on ESCs and azithromycin, and ideally also collect improved epidemiological data of patients and monitor failures to cure gonorrhoea with recommended treatment in Kyrgyzstan and globally. In Kyrgyzstan, hopefully this gonococcal AMR surveillance can be expanded outside the capital city Bishkek, i.e. more representative isolates examined from also additional regions of Kyrgyzstan. However, political support, additional funding and training in, for example, appropriate, quality assured sample collection, sample transportation, and gonococcal culture and AMR testing methodologies in additional Kyrgyz laboratories are essential for this expansion.

In general, it is crucial to implement quality-assured gonococcal AMR surveillance in also the neighbouring Central Asian countries as well as other former Soviet Union republics, where the gonococcal AMR surveillance has been exceedingly limited [55, 56] and restricted to limited surveillance in Russia, Belarus, and Ukraine [38–46]. Accordingly, among neighbouring Central-Asian countries of Kyrgyzstan, no data have been published from Tajikistan and Uzbekistan, and only very limited molecular typing data from 2008 to 2009 (48 isolates) in Kazakhstan [57]. Notably, the most common NG-MAST ST (ST1751; *n* = 21) in Kyrgyzstan according to our present study was reported from neighbouring Kazakhstan in 2008–2009 [57] and it was also the second most prevalent ST in Russia in 2017 [33]. Furthermore, ST807 represented by five isolates in Kyrgyzstan was the most common gonococcal NG-MAST ST in Russia in 2013–2018 [33] and it was also found in one isolate in Kazakhstan in 2008–2009 [57]. These results indicate some larger spread of these gonococcal genotypes in the Central Asian countries and in Russia. Worryingly, reported AMR from neighbouring China has been high, including resistance to ceftriaxone and internationally spreading ceftriaxone-resistant strains have been expanding in China [21, 58, 59]. Accordingly, the possible future import to Kyrgyzstan of ceftriaxone-resistant strains and multidrug-resistant gonococcal strains from China can not be excluded.

Using NG-MAST, the present study showed a diversified population of *N. gonorrhoeae* strains in Kyrgyzstan during 2012 and 2017 with 69 different NG-MAST STs. The high number of unique STs (*n* = 44, 63.8%) and STs that have not been described earlier (*n* = 52, 75.4%) may be associated with suboptimal diagnostics (only random gonorrhoea patients and/or isolates are identified), contact tracing (sexual contacts having the identical ST are not traced) and epidemiological surveillance (sexual transmission chains spreading a single ST are not identified or followed-up), STs evolved locally in Kyrgyzstan (STs are not previously described because no NG-MAST studies have previously been performed in the country) or imported from abroad. However, some main ST clusters caused by clonal spread of, e.g., G1751 (*n* = 21), G5488 (*n* = 12), and G436 (*n* = 8), were identified, which indicate several larger sexual transmission chains. To improve the resolution and accuracy of this molecular typing, it would be of interest to further examine the Kyrgyz strains with whole-genome sequencing (WGS) [60], which have not yet been applied on any gonococcal strains in Central Asia. Using WGS, the Kyrgyz gonococcal population can be compared to the strains spreading in European Union/European Economic Area (EU/EEA) [60, 61], other Eastern European countries such as Ukraine [46], and globally [62–67].

The limitations of the present study included that no information about patients’ sexual behaviour and their sexual partners, prescribed antimicrobial treatments, and treatment outcomes were available. Furthermore, no extragenital samples were taken (not routine practice in Kyrgyzstan, which may indicate some level of stigmatization regarding gonorrhoea, sexual practices and sexual orientations [6]), and isolates were only collected in the capital city Bishkek (only laboratory with appropriate culture facilities that was found), which represents 15.9% of the Kyrgyz population. However, the total coverage of investigated *N. gonorrhoeae* isolates to all nationally reported gonorrhoea cases was substantial, i.e. 8.8% (84/953) in 2012 and 15.6% (72/462) in 2017 (https://dgsen.kg), which allows our AMR results to appropriately inform refinements of the national Kyrgyz treatment guideline.

## Conclusions

*N. gonorrhoeae* isolates cultured in Kyrgyzstan in 2012 and 2017 showed a high genetic diversity and high levels of in vitro resistance particularly to antimicrobials internationally earlier recommended for gonorrhoea treatment. No in vitro resistance to ceftriaxone or spectinomycin was found, but a relatively high level of decreased susceptibility (MIC = 0.125 mg/L) to ceftriaxone (12.8%) was documented. Ceftriaxone, 500–1000 mg, in combination with azithromycin 2 g or doxycycline, particularly when chlamydial infection has not been excluded, should be recommended as empiric first-line treatment. Spectinomycin 2 g could be an alternative treatment, and given with azithromycin 2 g if pharyngeal gonorrhoea has not been excluded. Fluoroquinolones, aminoglycosides, benzylpenicillin, or tetracyclines should not be used for empiric treatment of gonorrhoea in Kyrgyzstan. Timely review and updating of and high compliance to national evidence-based gonorrhoea treatment guidelines that are based on quality-assured AMR data is imperative [3, 4, 25, 68]. Expanded and improved gonococcal AMR surveillance in Kyrgyzstan is crucial.

## Data Availability

The data that support the findings of this study are available from the corresponding author upon reasonable request.

## Abbreviations

AMR: Antimicrobial resistance
AZM: Azithromycin
CFM: Cefixime
CI: Confidence interval
CIP: Ciprofloxacin
CRO: Ceftriaxone
ECOFF: Epidemiological cut-off
EEA: European Economic Area
ESC: Extended-spectrum cephalosporin
EU: European Union
EUCAST: European Committee on Antimicrobial Susceptibility Testing
G: Genogroup
IM: intramuscular
IQR: Interquartile range
KAN: Kanamycin
MIC: Minimum inhibitory concentration
N: Number
NA: Not applicable
ND: Not determined
NG-MAST: *N. gonorrhoeae* multiantigen sequence typing
OR: Odds ratio
PEN: Benzylpenicillin
PPNG: Penicillinase-producing *Neisseria gonorrhoeae*
R: resistance
S: Susceptibility
SPC: Spectinomycin
ST: Sequence type
STI: Sexually transmitted infection
TET: Tetracycline
WGS: Whole genome sequencing
WHO: World Health Organization
